# Internal Endoconduit-Assisted Transfemoral TAVR in a Patient With Severely Diseased Iliofemoral Anatomy

**DOI:** 10.1016/j.jaccas.2026.109009

**Published:** 2026-06-27

**Authors:** Shinya Minami, Yasuhiro Ichibori, Satoshi Nakawatase, Naoki Mori, Osamu Iida

**Affiliations:** Cardiovascular Division, Osaka Keisatsu Hospital, Osaka, Japan

**Keywords:** internal endoconduit, percutaneous access, peripheral artery disease, transcatheter aortic valve replacement, transfemoral access

## Abstract

**Background:**

The internal endoconduit technique may facilitate transfemoral transcatheter aortic valve replacement (TAVR) in complex vascular anatomies.

**Case Summary:**

A 71-year-old woman with symptomatic severe aortic stenosis and diffusely narrowed iliac and femoral arteries was considered unsuitable for transfemoral access. A staged, internal endoconduit-assisted transfemoral TAVR was performed. First, overlapping VIABAHN covered stent grafts were deployed to create an arterial conduit in iliofemoral arteries. Two weeks later, a 26-mm Evolut FX valve was successfully implanted. Hemostasis was successfully achieved using Perclose ProStyle sutures and an additional stent graft.

**Discussion:**

The internal endoconduit technique in TAVR has not been previously described in detail. A preprocedural ex vivo simulation was performed to devise an optimal strategy, enabling the safe execution.

**Take-Home Message:**

The internal endoconduit technique represents a viable alternative for overcoming hostile vascular anatomy during transfemoral TAVR; nonetheless, important considerations remain, such as the interval before TAVR, the graft puncture site, and sheath selection.

## Visual Summary

**Figure:**
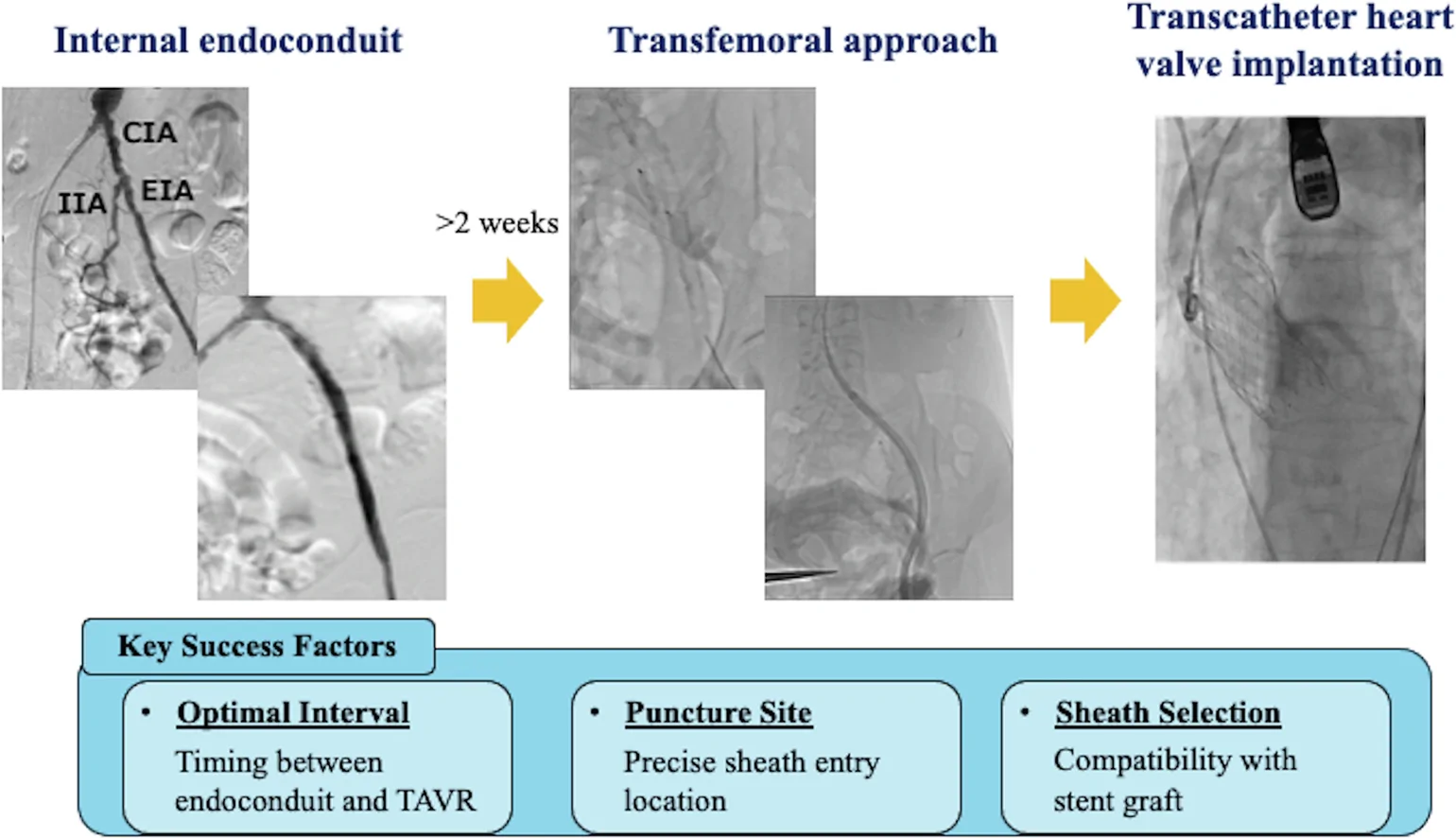

**Visual Summary:**
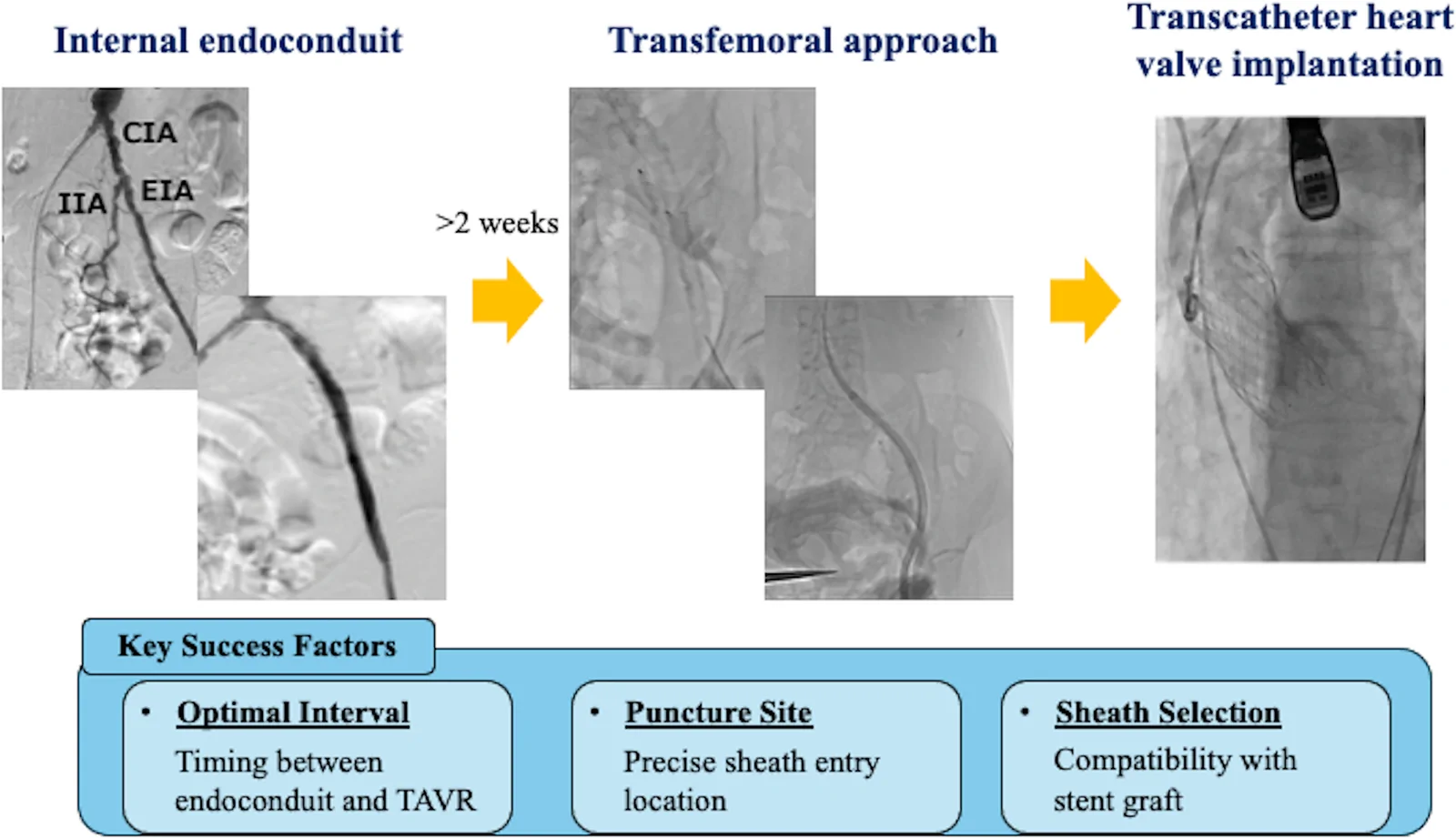
Overcoming Hostile Vascular Anatomy Using Internal Endoconduit Technique CIA = common iliac artery; EIA = external iliac artery; IIA = internal iliac artery; TAVR = transcatheter aortic valve replacement.

## History of Presentation

A 71-year-old woman with symptomatic severe aortic stenosis was referred to our institution for transcatheter aortic valve replacement (TAVR). She presented with progressively worsening exertional dyspnea. Preoperative transthoracic echocardiography revealed a severely stenotic tricuspid aortic valve with a valve area of 0.7 cm^2^, a peak velocity of 3.6 m/s, and a preserved left ventricular ejection fraction of 77%. Her surgical risk was classified as low to intermediate, with a Society of Thoracic Surgeons score of 3.2% and a EuroSCORE II of 16.9%. However, she was deemed frail, with a Clinical Frailty Scale score of 5 and a body mass index of only 13.2, reflecting significant sarcopenia and undernutrition.

## Past Medical History

Her past medical history was notable for severe chronic obstructive pulmonary disease, chronic kidney disease (estimated glomerular filtration rate was 32 mL/min/1.73 m^2^), lower extremity peripheral artery disease, and iron deficiency anemia. She had no prior history of cardiac surgery or percutaneous coronary intervention.

**Take-Home Messages**

• The internal endoconduit technique can facilitate transfemoral transcatheter aortic valve replacement in patients with severely diseased iliofemoral arteries when no other access route is feasible.
• A staged approach with careful sheath selection, puncture site choice, and preplaced sutures may optimize procedural safety and hemostasis.

## Investigations

**Figure 1:**
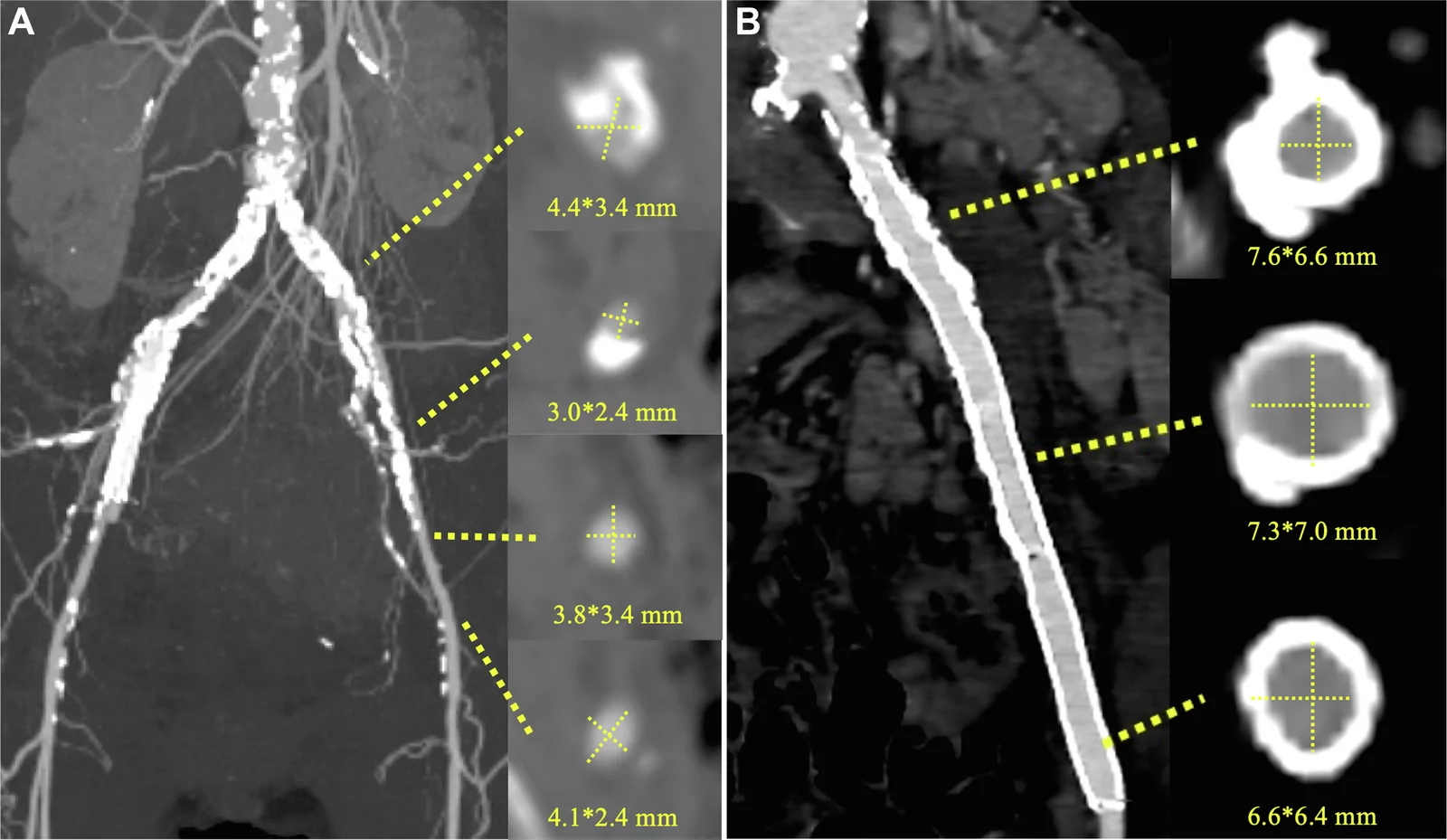
CT Angiography of the Left Access Artery Before and After the Internal Endoconduit (A) MIP of the preoperative CT angiogram reveals heavily calcified and severely stenotic iliac and common femoral arteries. The reconstructed cross-sectional images perpendicular to the center line of the left iliofemoral artery demonstrate markedly reduced luminal diameters, ranging from 2.4 to 4.4 mm. (B) MIP of the postoperative CT angiogram demonstrates a stent graft–supported endoconduit along the left iliac and common femoral arteries. Cross-sectional images of the respective portions show marked luminal expansion, with diameters ranging from 6.4 to 7.6 mm, which most likely indicated rupture of the native iliac artery after stent graft deployment and balloon angioplasty. CT = computed tomography; MIP = maximum intensity projection.

Contrast-enhanced computed tomography revealed tricuspid aortic valve morphology with calcification on all 3 cusps. The anatomy of the aortic root was suitable for TAVR; however, transfemoral access routes were deemed unsuitable. Both iliac and common femoral arteries showed extensive atherosclerotic changes and diffusely narrowed with luminal diameters <4 mm, thus precluding the insertion of a large-caliber delivery system or sheath (Figure 1A). In addition, both subclavian arteries measured approximately 4 mm in diameter, which also precluded the insertion of a large-caliber delivery system or sheath. Transcarotid access was not considered anatomically ideal owing to the absence of both anterior and posterior communicating arteries on magnetic resonance angiography of the cerebral and neck vessels. Transcaval access was anatomically unfeasible due to diffuse calcification of the abdominal aorta in the segment below the renal veins where it is adjacent to the inferior vena cava (Table 1).

**Table 1:** Internal Endoconduit Creation for Transfemoral TAVR

Timeline	Events
Date of submission/day 1	A 71-year-old woman presented with NYHA functional class III symptoms. Echocardiography showed severe aortic stenosis.
Day 7	Contrast-enhanced computed tomography demonstrated extensive atherosclerotic disease and diffuse narrowing of both iliac and subclavian arteries, with luminal diameters measuring <4 mm, thereby precluding the insertion of a large-caliber delivery system or sheath.
Day 9	Magnetic resonance angiography of the cerebral vessels revealed the absence of both anterior and posterior communicating arteries.
Day 29	The internal endoconduit was created under general anesthesia. Two covered stent grafts were deployed in an overlapping fashion from the left common iliac artery to the common femoral artery.
Day 43	The patient underwent transfemoral transcatheter aortic valve replacement without any complications.
Day 97	She presented to the outpatient clinic in good condition, without any symptoms in the lower extremities.

## Management (Medical/Interventions)

Because of the presence of chronic lung disease, severe frailty, and the patient's refusal of open surgical approaches, alternative access routes such as transapical and transaortic approaches as well as surgical aortic valve replacement were considered unfeasible. Finally, after heart team discussion including cardiologists, cardiovascular surgeons, and anesthesiologists and obtaining approval from the institutional ethics review board, we decided to perform a 2-stage internal endoconduit-assisted transfemoral TAVR. The left iliofemoral artery was selected for the access because the patient had a history of bare-metal stent implantation in the contralateral (right) external iliac artery for peripheral artery disease.

**Figure 2:**
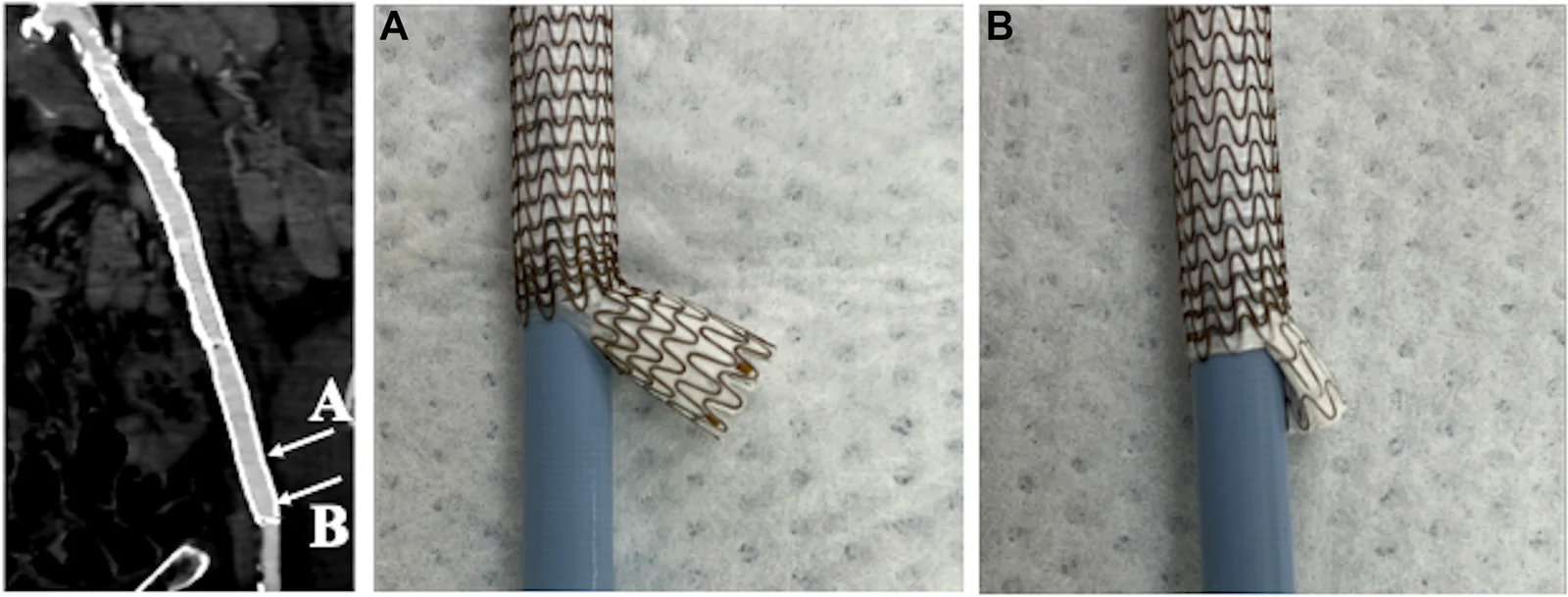
Impact of Puncture Sites on the VIABAHN (A) More central vs (B) closer to the distal edge. The risk of distal-edge displacement was comparable between sites, whereas distal-edge deformation was higher with puncture at (B).

**Figure 3:**
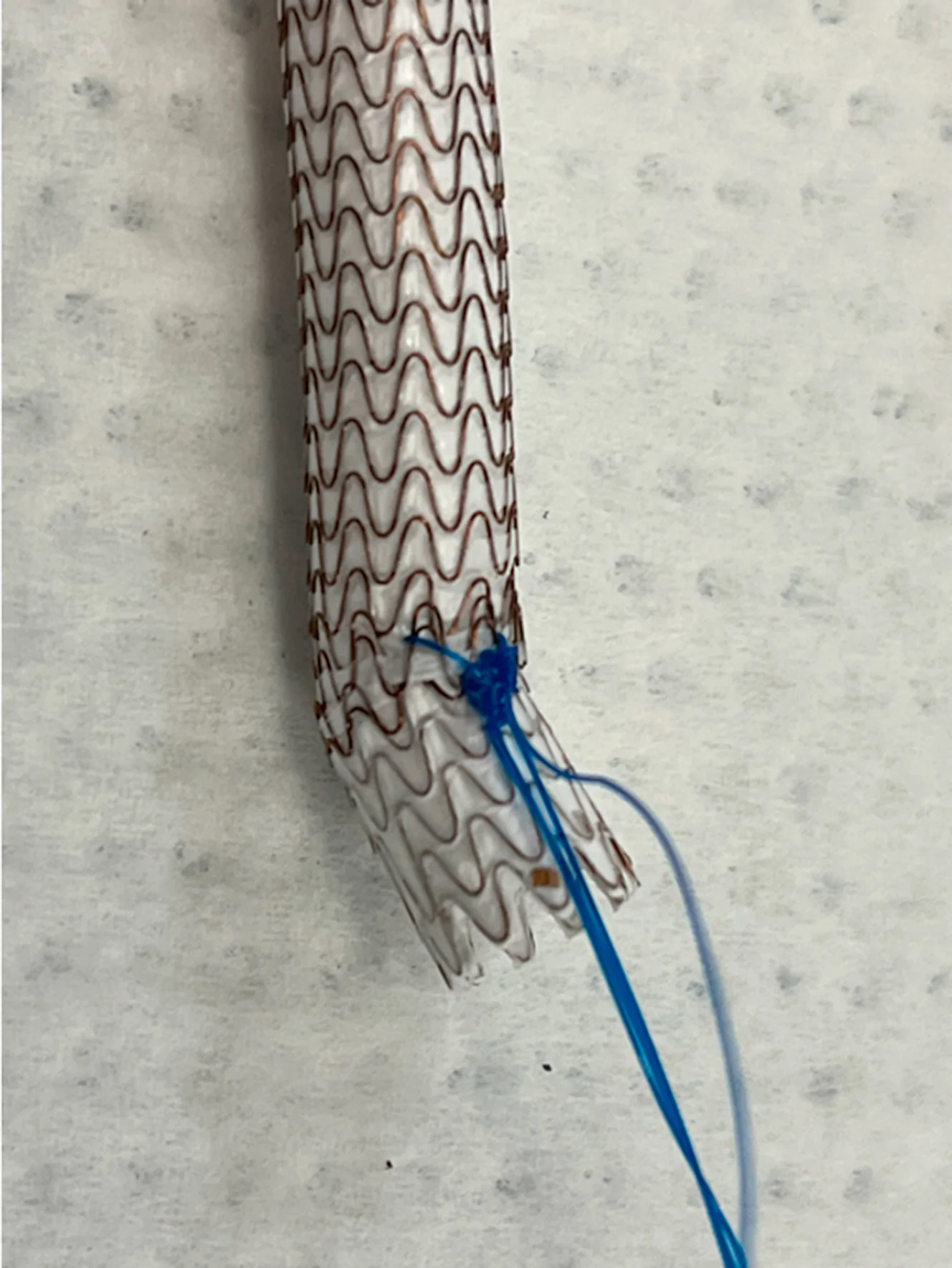
Preplacement of Perclose ProStyle Sutures and ePTFE Apposition at the Sheath Entry Site Two preplaced sutures gathered the ePTFE covering tightly around the entry site, which is expected to facilitate enough hemostasis. ePTFE = expanded polytetrafluoroethylene.

A preprocedural ex vivo simulation was performed. We observed that when inserting large-caliber sheaths into an 8-mm-diameter GORE VIABAHN (W. L. Gore & Associates) covered stent graft, the use of an 18-F DRYSEAL sheath (W. L. Gore & Associates) was superior to a 14-F eSheath (Edwards Lifesciences). The stepped transition at the junction of the eSheath made it more prone to catching on the nitinol structure underlying the VIABAHN's expanded polytetrafluoroethylene layer, increasing the risk of conduit damage. In contrast, the smoother profile of the DRYSEAL sheath allowed safer navigation through the stent graft. Moreover, our bench experiments highlighted that puncturing the VIABAHN near its distal edge increased the risk of deformation and collapse, reinforcing the importance of selecting a more central puncture site along the stent graft body to preserve its structural integrity (Figure 2). In addition, we found that preplacing 2 Perclose ProStyle (Abbott) sutures before sheath insertion was considered sufficient to approximate the expanded polytetrafluoroethylene layer and nitinol structure of the VIABAHN and close the puncture created by the sheath to achieve hemostasis (Figure 3).

In the first stage, the internal endoconduit was created under general anesthesia. Initially, left internal iliac artery embolization was performed using the Amplatzer Vascular Plug (Abbott). Because the contralateral internal iliac artery was patent, this plug placement was crucial to prevent retrograde bleeding via cross-pelvic collateral pathways such as the rectal arterial plexus, conceptually analogous to preventing a type II endoleak during endovascular aneurysm repair.^1^ Subsequently, 2 GORE VIABAHN covered stent grafts (8 mm in diameter with 100 mm in length and 10 mm in diameter with 100 mm in length) were deployed in an overlapping fashion from the left common femoral artery to the common iliac artery. Postdeployment balloon angioplasty was performed with a 9-mm noncompliant balloon to expand the conduit sufficiently to accommodate a large-bore sheath (Figure 1B).

**Figure 4:**
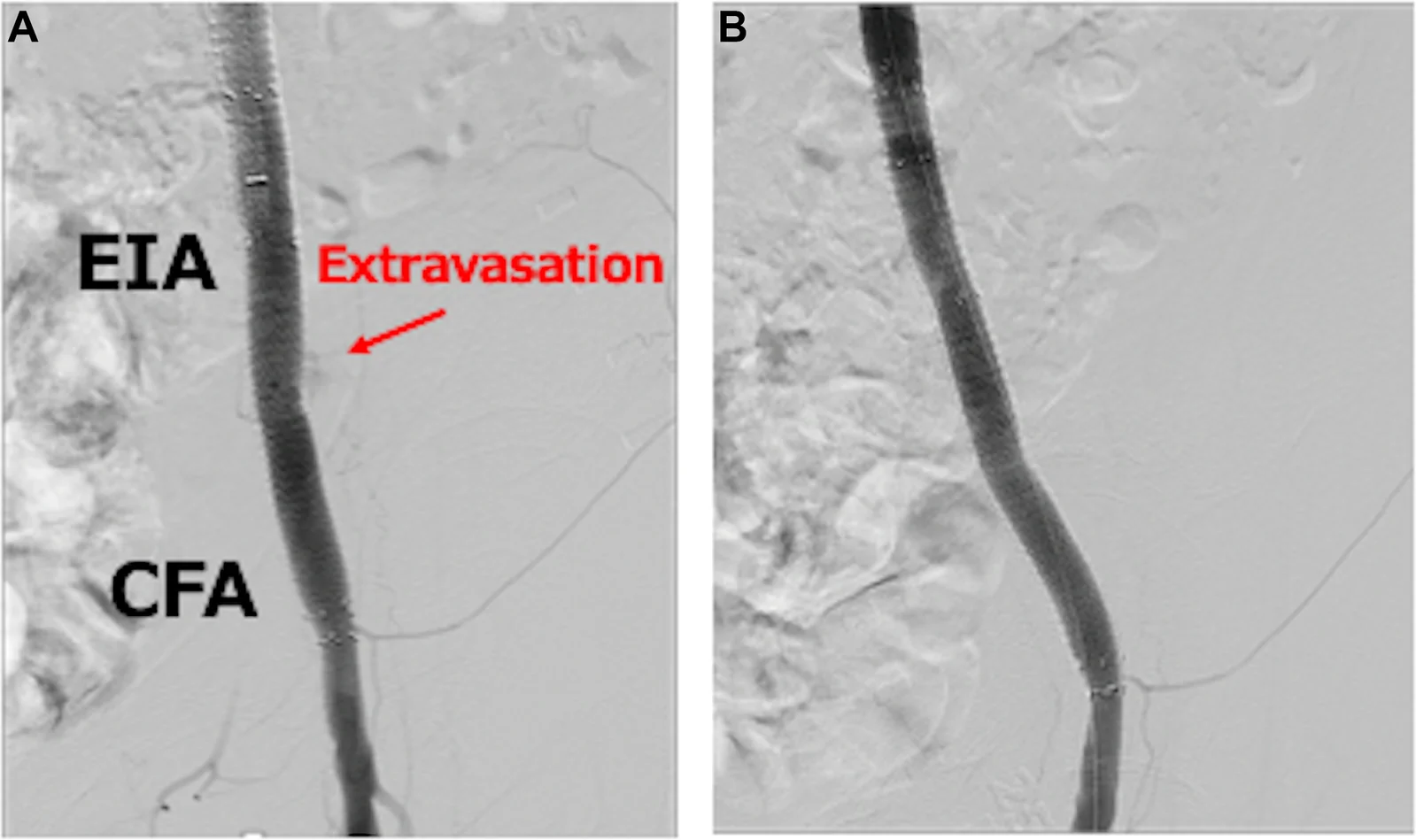
Final Iliofemoral Artery Angiography (A) After fastening the prepositioned sutures, trivial access site bleeding persisted. (B) Complete hemostasis was obtained with an additional stent-graft implantation. CFA = common femoral artery; EIA = external iliac artery.

Two weeks later, in the second stage, TAVR was performed. Vascular access was obtained via the previously constructed endoconduit using an 18-F DRYSEAL introducer sheath after preplacing 2 Perclose ProStyle sutures. A 26-mm Evolut FX (Medtronic) self-expanding transcatheter valve was successfully delivered and deployed without complication. Aortography confirmed appropriate valve position with no evidence of paravalvular leak or coronary obstruction. Hemostasis at the access site was performed using prepositioned sutures as initially planned. However, trivial bleeding was observed on angiography. Although manual compression was considered as an option, an additional VIABAHN stent graft was deployed, achieving complete hemostasis (Figure 4). No deformation or collapse of the stent grafts was observed under fluoroscopy surveillance. The patient had an uneventful postoperative course and was discharged in stable condition.

## Discussion

TAVR has emerged as a minimally invasive treatment for severe aortic stenosis.^2^ Currently, because of its minimally invasive nature and low complication rates, the transfemoral approach is considered the first-line strategy. However, in patients with severe calcification, tortuosity, or stenosis of the iliofemoral arteries, this approach can be challenging.^3^ In such cases, alternative access routes such as transcarotid or subclavian artery approaches are considered, although some patients are not suitable candidates for these routes either. Among these, the transcaval TAVR is an emerging alternative for hostile anatomies. However, its applicability may be limited in certain cases due to unfavorable aortic anatomy, such as severe calcification at the aortocaval interface. In addition, the potential limitations in managing procedural complications without access to the transfemoral route should be taken into consideration when selecting the optimal access strategy.^4^

Recently, intravascular lithotripsy (IVL) has become a valuable tool for modifying severe calcification to facilitate transfemoral access, often combined with lower-profile delivery systems and bailout stenting. However, IVL primarily addresses calcification rather than the absolute vessel size. In our patient, the iliofemoral arteries were not only heavily calcified but also diffusely narrow (<4 mm). Attempting to expand such small-caliber vessels, even after IVL, carries a prohibitive risk of uncontrolled rupture.

The internal endoconduit technique has emerged as a viable solution for enabling transfemoral access in patients undergoing thoracic endovascular aortic repair with severely diseased iliofemoral arteries in recent years.^5^ This technique involves the endovascular deployment of a stent graft to create a conduit through which large-caliber sheaths can be safely advanced. It has demonstrated a low rate of early mortality and access-related complications, even in vessels measuring <5 mm in diameter. Van Bogerijen et al^6^ conducted a single-center, retrospective study comparing the retroperitoneal open iliac conduit (n = 23) with the internal endoconduit technique (n = 16). They found that iliofemoral complications occurred in 26.1% of patients in the open iliac conduit group, whereas the rate was lower in the internal endoconduit group (12.5%). This promising safety profile makes it an attractive option for selected patients undergoing TAVR with hostile access anatomy. The use of the internal endoconduit technique during TAVR has been reported only in a few cases.^7^, ^8^, ^9^ However, unlike these reports, our case demonstrates a highly novel approach where an internal endoconduit was made over a long segment from the common iliac artery to the common femoral artery, and direct puncture into the conduit was performed. To the best of our knowledge, this specific long-segment, direct-puncture endoconduit strategy for TAVR has not been previously described.

In our case, the internal endoconduit technique was applied in a 2-stage manner. VIABAHN stent grafts were first deployed to establish a conduit, followed by delayed TAVR after confirming the conduit integrity. Timaran et al^10^ reported that staging the procedure by waiting a few weeks after endoconduit creation allows vascular healing, thereby promoting perigraft incorporation and more secure stent-graft fixation. In addition, the preprocedural ex vivo simulation informed our procedural strategy. The smoother profile of the DRYSEAL sheath allowed safer navigation through the stent graft. Puncturing the VIABAHN at the more central site along the stent graft body works to preserve its structural integrity. We found that preplacing 2 Perclose ProStyle sutures before sheath insertion was sufficient to achieve substantial hemostasis. However, to obtain complete hemostasis, additional manual compression or deployment of an additional stent graft may be required.

## Conclusions

The internal endoconduit technique represents a viable alternative for enabling transfemoral TAVR in patients with unsuitable iliofemoral anatomy and no other feasible alternative access routes.

## Funding Support and Author Disclosures

Dr Ichibori is a proctor for Medtronic Japan and receives lecture fees from Medtronic Japan. Dr Iida has received lecture fees from Medtronic Japan and W. L. Gore & Associates. All other authors have reported that they have no relationships relevant to the contents of this paper to disclose.

## Appendix

Video 1 Preprocedural Simulation and Internal Endoconduit Technique for Transfemoral TAVR The video demonstrates the planning and procedural steps of an internal endoconduit technique for transfemoral TAVR. After computed tomography–based anatomical assessment, a preprocedural ex vivo simulation was performed to assess sheath insertion through the stent-graft conduit and the optimal preclosure sutures. The simulation informed procedural planning and facilitated the safe execution of transfemoral TAVR in the setting of severely diseased iliofemoral access. TAVR = transcatheter aortic valve replacement.
